# The predictive value of cord blood 25-hydroxyvitamin D and vitamin A levels for bronchopulmonary dysplasia in preterm infants

**DOI:** 10.3389/fped.2026.1948901

**Published:** 2026-10-28

**Authors:** Xiaoxia Zhang, Chunmei Wan, Xiaomei Wang, Lan Jin, Shanshan Zhao, Xi Wang, Wenbin Dong

**Affiliations:** 1 Department of Neonatology, Sichuan Provincial Clinical Medical Research Center for Birth Defects, Affiliated Hospital of Southwest Medical University, Sichuan, China; 2 Department of Neonatology, Southern Central Hospital of Yunnan Province, The First People's Hospital of Honghe State, Yunnan Province, China

**Keywords:** 25-hydroxyvitamin D, bronchopulmonary dysplasia, cord blood, C-reactive protein, interleukin-6, preterm infants, procalcitonin, vitamin A

## Abstract

**Background:**

Despite substantial progress in the treatment of bronchopulmonary dysplasia (BPD), its overall incidence remains high, with increasing rates of moderate-to-severe cases. Reliable early biomarkers are urgently needed to facilitate timely intervention and optimize long-term prognosis.

**Methods:**

This prospective cohort study enrolled 144 preterm infants with gestational age ≤32 weeks admitted to the neonatal intensive care unit from March 2021 to December 2024. Umbilical arterial blood was collected to detect 25(OH)D, vitamin A (VA), IL-6, PCT, and CRP levels. Infants were classified into BPD (*n* = 63) and non-BPD (*n* = 81) groups, and the BPD group was further stratified into Grade I, II, and III subgroups according to oxygen requirement and respiratory support. Group differences in biomarker levels and clinical variables were analyzed. Univariate and multivariate logistic regression models were constructed to identify independent BPD predictors. ROC curves, DeLong test, NRI, and IDI were applied to evaluate incremental predictive performance.

**Results:**

Cord blood 25(OH)D and VA were significantly decreased, while PCT was elevated in BPD infants (all *P* < 0.05). Within BPD subgroups, 25(OH)D gradually decreased and IL-6 increased with disease severity. Multivariate regression confirmed that cord blood 25(OH)D remained an independent protective factor for BPD after adjustment for gestational age, birth weight, and caffeine exposure (OR = 0.854, *P* < 0.001), whereas VA showed no independent predictive significance. The baseline clinical model yielded an AUC of 0.784. The addition of 25(OH)D significantly improved predictive efficacy to AUC = 0.857 (ΔAUC = 0.071, *P* = 0.001), with robust NRI and IDI improvements (both *P* < 0.001). Further inclusion of VA produced no additional predictive benefit.

**Conclusion:**

Umbilical arterial 25(OH)D is a stable independent predictive biomarker for BPD and provides valuable incremental predictive value beyond conventional clinical parameters, supporting early risk stratification in preterm infants, whereas VA exhibits limited predictive utility.

## Introduction

Over five decades ago, Northway et al. first characterized bronchopulmonary dysplasia (BPD) (1). Despite more than 50 years of scholarly progress, the incidence of BPD has not declined (2), and no novel therapeutic strategy has yet been successfully translated into routine clinical practice (2, 3). BPD remains among the most prevalent and severe complications affecting preterm infants and constitutes a major determinant of mortality and adverse long-term neurodevelopmental and pulmonary outcomes in this vulnerable population (4, 5). Although the pathogenesis of BPD remains incompletely understood, robust clinical and preclinical evidence identifies hyperoxia-induced lung injury, intrauterine or postnatal infection, and dysregulated inflammatory responses as key drivers of aberrant alveolar and vascular development in affected preterm infants (5, 6). The pathological features of BPD mainly include abnormal lung function, delayed motor development, and feeding difficulties, accompanied by varying degrees of pulmonary fibrosis and pulmonary leukocyte infiltration, which significantly impair growth and neurodevelopment in affected infants (7–9). Accordingly, early and accurate diagnosis of BPD holds critical significance for guiding the formulation of individualized clinical treatment regimens and optimizing long-term prognosis.

In recent years, umbilical cord blood (UCB) testing has emerged as a prominent focus in perinatal biological research. Compared with postnatal blood testing in neonates, UCB testing offers distinct advantages: it not only circumvents interference from neonatal physiological fluctuations on assay results but also reduces the need for invasive procedures, thereby significantly alleviating iatrogenic distress. Furthermore, UCB is enriched with a diverse array of disease-related biomarkers, rendering it an ideal sample source for early risk stratification and mechanistic investigations of developmental lung disorders (10, 11). Studies have shown that 25-hydroxyvitamin D [25(OH)D] is the primary active metabolite of vitamin D (VD). As a fat-soluble vitamin, it plays an important role in inhibiting pulmonary fibrosis, exerting anti-inflammatory effects, promoting angiogenesis, alveolar development, and lung tissue repair (9–12). Vitamin A (VA) is an essential nutrient for maintaining vital bodily functions, playing roles in promoting growth and development, immune regulation, and resistance to infection (13, 14). Interleukin-6 (IL-6) is a multifunctional pro-inflammatory cytokine composed of 184 amino acids, playing pleiotropic roles in inflammatory responses, immune regulation, and hematopoiesis. IL-6 can accelerate the progression of pulmonary inflammation. Studies have found that in children with lung infections, vitamin A (VA) levels are significantly reduced, while IL-6 levels are markedly elevated (15, 16). The release of inflammatory mediators is associated with pulmonary capillary injury and an increased risk of BPD (17). Procalcitonin (PCT), a biomarker for inflammatory mediators, typically rises during the early phase of disease with high specificity; its serum levels correlate with the severity of tissue injury (18). C-reactive protein (CRP) is an acute-phase reactant. Studies indicate that umbilical cord blood levels of CRP, procalcitonin (PCT), and interleukin-6 (IL-6) are significantly elevated in preterm infants with intrauterine infection compared to non-infected controls, suggesting that these biomarkers play critical roles in the pathogenesis of intrauterine infection (19).

Data regarding the association between umbilical cord blood levels of 25-hydroxyvitamin D [25(OH)D], vitamin A (VA), and inflammatory markers (IL-6, PCT, CRP) and the subsequent development of bronchopulmonary dysplasia (BPD) in preterm infants remain scarce. Therefore, this study aims to evaluate the correlations between these biomarkers and BPD incidence, thereby providing a theoretical foundation for early risk stratification and intervention strategies.

## Materials and methods

### Study design and population

This prospective, single-center controlled study included 144 preterm infants (gestational age ≤32 weeks) born at our hospital's Department of Obstetrics and admitted to the Neonatal Intensive Care Unit (NICU) between March 2021 and December 2024. BPD was diagnosed following the standardized classification system proposed by the 2018 NICHD International BPD Consensus Conference (20). Inclusion criteria were as follows: (1) delivery at our institution with umbilical artery blood collected at birth; (2) gestational age at birth ≤32 weeks; and (3) hospitalization for at least 28 days. Exclusion criteria included: (1) the presence of structural heart malformations, inherited metabolic errors, chromosomal disorders, multiple congenital anomalies, or other serious disorders; (2) discontinuation of the study due to death, voluntary withdrawal, or other reasons; (3) congenital pulmonary hypoplasia or structural lung malformations; (4) hypothyroidism; (5) hepatic, renal, or other major organ dysfunction; (6) severe intraventricular hemorrhage or cerebral developmental abnormalities; (7) maternal conditions potentially affecting VD metabolism during pregnancy, including metabolic bone disease, thyroid dysfunction, liver or kidney disease, or use of antiepileptic/anticonvulsant medications; and (8) incomplete clinical records. Ethical approval was given by the Ethics Committee of our institution (Approval No.: HY2021LLSC-110), and the study was carried out per the principles of the Declaration of Helsinki (Figure 1).

**Figure 1:**
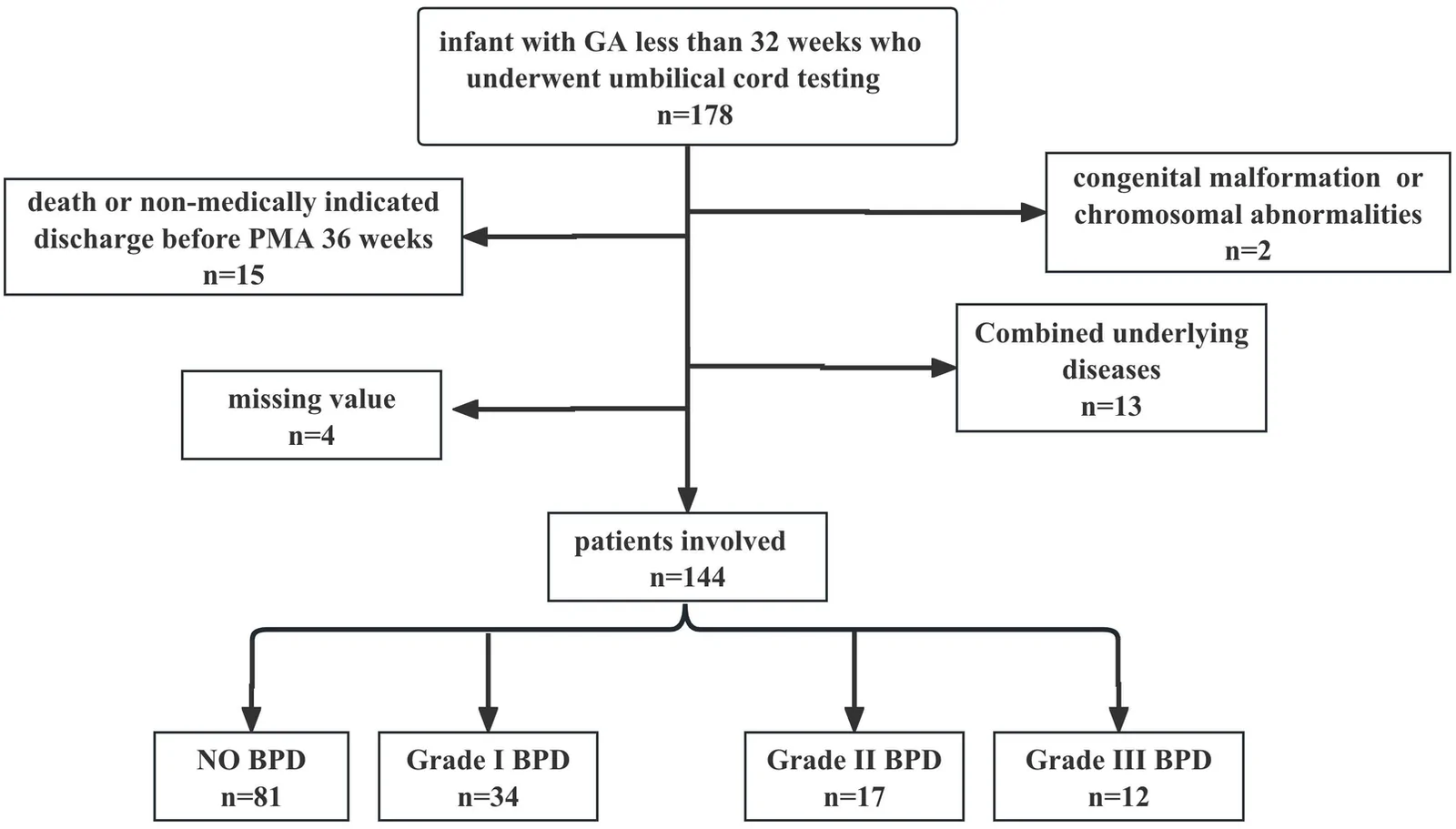
Patient selection flowchart.

### Collection of cord blood

Cord blood, as the earliest and most readily accessible biological sample from newborns, offers advantages such as convenient collection and non-invasiveness (10). During delivery, for preterm infants not requiring immediate resuscitation and eligible for delayed umbilical cord clamping (DCC), trained clinical personnel perform bilateral umbilical artery puncture prior to cord clamping. Umbilical artery blood is selected because it consists of fetal venous blood returning from the fetus to the placenta—thereby directly reflecting fetal circulatory physiology and endogenous biological profiles—and thus provides superior physiological fidelity for evaluating biomarkers associated with fetal lung development. Each puncture yields 5 mL of whole blood collected in pre-chilled, sterile, EDTA-free tubes. In cases requiring neonatal resuscitation, umbilical artery puncture is performed at the placental end by midwives uninvolved in the resuscitation team, and specimens are immediately transported under controlled conditions to the laboratory for centrifugation, serum separation, and cryopreservation. Serum samples not assayed within 24 h are stored according to a tiered protocol: protected from light at room temperature (20–25 °C) for ≤24 h; refrigerated at 2–8 °C for ≤7 days; frozen at −20 °C for ≤12 months; or stored long-term at −80 °C for ≤14 months. All specimens are assigned a unique, anonymized alphanumeric code to ensure unambiguous identification and full traceability across all analytical stages.

### Detection of 25(OH)D, VA

The technical parameters employed in this study were strictly adhered to international laboratory protocols, including but not limited to sample dilution factors and specifications for high-performance liquid chromatography (HPLC) columns (e.g., column type and dimensions). Liquid chromatography-tandem mass spectrometry (LC-MS/MS) is internationally recognized as the gold standard for vitamin analysis and represents the preferred method for clinical quantification of small biochemical molecules, owing to its high accuracy, excellent specificity, high throughput, and superior sensitivity (21). LC-MS/MS is an analytical technique that employs a mass analyzer to determine the mass-to-charge ratio (m/z) of charged particles. Prior to clinical quantification of 25(OH)D and VA by LC-MS/MS, the assay must undergo full method validation in accordance with established guidelines (e.g., CLSI C62-A); only after successful validation may it be implemented for routine clinical sample analysis. The instrumentation used in this study is the AB Sciex QTRAP™ 4500 LC-MS/MS system (Manufacturer: AB Sciex LLC; local technical support provided by Jiangsu Haosi Biotechnology Co., Ltd.).

### Measurement of CRP, PCT, and IL-6

An additional 5 mL of umbilical artery blood was collected and allowed to coagulate naturally at room temperature for 10–20 min, followed by centrifugation (3,000 rpm, 15 min) to separate the serum. Quantification of inflammatory biomarkers, including IL-6, PCT, and CRP, was performed using chemiluminescence immunoassay (CLIA) technology. All measurements were conducted using a DXI800 fully automated CLIA analyzer (Beckman Coulter, Inc., USA), an established platform known for high-sensitivity immunoassays. Analytical procedures strictly followed the manufacturer's standardized operating protocols to ensure reproducibility and accuracy.

### Statistical analysis

This study used SPSS 26.0 statistical software for data analysis. Continuous variables were assessed for normality; those conforming to a normal distribution were presented as mean ± standard deviation, and group comparisons were performed using independent-samples *t*-tests. Non-normally distributed variables were expressed as median (interquartile range) [*M*(Q1, Q3)], with between-group comparisons conducted using the Mann–Whitney *U* test, and multi-group comparisons analyzed using the Kruskal–Wallis test. Categorical variables were summarized as frequency (percentage) [*n* (%)], and group comparisons were performed using chi-square tests (*χ*^2^ tests). Receiver operating characteristic (ROC) curves were constructed to calculate the area under the curve (AUC) for evaluating the predictive value of umbilical cord blood 25(OH)D, VA, and other biomarkers for BPD; optimal cutoff values for each indicator were determined using the Youden index. Multivariate logistic regression was employed to identify independent risk factors associated with BPD. When complete separation occurred in the multivariate model, Firth's penalized logistic regression was applied. DeLong's test was used to compare AUCs between nested models, and net reclassification improvement (NRI) and integrated discrimination improvement (IDI) were calculated to assess the incremental predictive value of newly added indicators. All statistical tests were two-tailed, and differences were considered statistically significant at *P* < 0.05.

## Results

### Cord blood concentrations of IL-6, PCT, CRP, 25(OH)D_3_, and vitamin A (VA) in the two groups

Intergroup comparisons of biomarker levels were conducted utilizing the M-W *U* test. As shown in Table 1, differences in CRP and IL-6 levels between the non-BPD and BPD groups did not reach statistical significance (*P* > 0.05). However, significant differences were identified for three key biomarkers: PCT concentrations were markedly higher in the BPD group compared to the control group (*P* < 0.05), while 25(OH)D and VA levels were significantly lower in the BPD group (*P* < 0.05). These findings specify a potential association between these specific biomarkers and the pathogenesis of BPD.

**Table 1:** Comparison of PCT, CRP, IL-6, 25 (OH)D, and VA levels in UCB at birth between the two groups.

Groups	Sample size	IL-6 (pg/mL)	PCT (ng/mL)	CRP (mg/L)	25-(OH)D (ng/mL)	VA (ng/mL)
Control group	81	104.69 (47.27, 215.98)	0.21 (0.17, 0.29)	0.50 (0.50, 0.50)	29.00 (20.63, 35.30)	206.00 (152.00, 288.00)
BPD group	63	132.42 (23.25, 664.20)	0.37 (0.27, 0.80)	0.50 (0.50, 0.98)	15.40 (12.60, 20.08)	150.00 (118.00, 193.00)
*Z*		−1.248	−5.431	−1.452	−6.657	−3.939
*P*		0.212	<0.001	0.146	<0.001	<0.001

### IL-6, PCT, CRP, 25(OH)D, and VA concentrations in umbilical cord blood (UCB) at birth across groups stratified by BPD severity

Differences in various indicators across BPD severity classifications [Grade I, II, and III (IIIa + IIIb)] were analyzed using the K-W test. Initial results showed no statistically significant differences in CRP and VA levels among the different BPD severity grades (*P* > 0.05). In contrast, IL-6, PCT, and 25(OH)D levels varied significantly across the severity groups (*P* < 0.05). *Post hoc* pairwise comparisons, adjusted using the Bonferroni correction for multiple testing, revealed several notable findings: IL-6 concentrations were significantly lower in Grade I patients compared to those with Grade III BPD (*P* < 0.05); PCT levels in Grade I were markedly lower than in Grade II patients (*P* < 0.05); and 25(OH)D levels revealed an inverse relationship with disease severity, being significantly higher in Grade I than in Grade III patients (*P* < 0.05) (Table 2). These results suggest a potential link between specific biomarker profiles and disease progression, with distinct biochemical patterns corresponding to different stages of BPD severity.

**Table 2:** Comparison of IL-6, PCT, CRP, 25 (OH)D, and VA levels in UCB at birth in different degrees of BPD groups.

BPD level	Sample size	IL-6 (pg/mL)	PCT (ng/mL)	CRP (mg/L)	25-(OH)D (ng/mL)	VA (ng/mL)
Grade I	34	86.66 (9.38, 374.45)	0.33 (0.26, 0.59)	0.50 (0.50, 1.02)	16.00 (14.08, 21.43)	153.00 (117.50, 196.75)
Grade II	17	132.42 (88.60, 1,010.25)	0.80 (0.35, 4.23)	0.50 (0.50, 3.70)	15.10 (12.25, 18.16)	154.00 (118.50, 222.00)
Grade III	12	278.36 (189.82, 1,116.25)	0.37 (0.21, 0.63)	0.50 (0.50, 0.50)	11.55 (9.90, 16.50)	143.00 (114.25, 171.00)
*H*		7.610	8.216	1.800	11.354	0.775
*P*		0.022	0.016	0.407	0.003	0.679

### Univariate analysis of factors associated with BPD occurrence

Statistical analysis of differences in baseline characteristics between the BPD and control groups is presented in Table 3. The BPD group exhibited significantly higher proportions of infants with gestational age <30 weeks, birth weight <1,500 g, perinatal asphyxia, placental abnormalities, surfactant (PS) administration, caffeine therapy, and invasive mechanical ventilation compared with the control group (all *P* < 0.05).

**Table 3:** Univariate analysis of factors associated with BPD occurrence.

Indicators		Control group	BPD group	*χ* ^2^	*P*
Sex	Male	47 (58.02)	42 (66.67)	1.121	0.290
	Female	34 (41.98)	21 (33.33)		
Gestational age	<30 weeks	2 (2.47)	24 (38.10)	30.400	<0.001
	≥30 weeks	79 (97.53)	39 (61.90)		
Weight	<1,500 g	19 (23.46)	41 (65.08)	25.259	<0.001
	≥1,500 g	62 (76.54)	22 (34.92)		
Number of fetuses	1	60 (74.07)	50 (79.37)	2.505	0.286
	2	18 (22.22)	13 (20.63)		
	3	3 (3.70)	0 (0.00)		
Birth asphyxia	No	58 (71.60)	22 (34.92)	19.314	<0.001
	Yes	23 (28.40)	41 (65.08)		
Maternal comorbidities	No	32 (39.51)	30 (47.62)	0.951	0.329
	Yes	49 (60.49)	33 (52.38)		
Prenatal hormone use	No	46 (56.79)	38 (60.32)	0.181	0.670
	Yes	35 (43.21)	25 (39.68)		
Premature rupture of membranes (time)	No	64 (79.01)	50 (79.37)	0.003	0.959
	Yes	17 (20.99)	13 (20.63)		
Abnormal placenta	No	41 (50.62)	16 (25.40)	9.425	0.002
	Yes	40 (49.38)	47 (74.60)		
Use of PS	No	40 (49.38)	15 (23.81)	9.818	0.002
	Yes	41 (50.62)	48 (76.19)		
Use of caffeine	No	36 (44.44)	7 (11.11)	18.800	<0.001
	Yes	45 (55.56)	56 (88.89)		
Invasive mechanical ventilation	No	39 (48.15)	16 (25.40)	7.770	0.005
	Yes	42 (51.85)	47 (74.60)		
Non-invasive mechanical ventilation	No	16 (19.75)	19 (30.16)	2.086	0.149
	Yes	65 (80.25)	44 (69.84)		
Vitamin D supplementation	No	7 (8.64)	8 (12.70)	0.625	0.429
	Yes	74 (91.36)	55 (87.30)		
Vitamin A supplementation	No	55 (67.90)	38(60.32)	0.891	0.345
	Yes	26(32.10)	25(39.68)		

### Multivariate binary logistic regression analysis of independent risk factors associated with BPD

To systematically evaluate the independent associations of umbilical cord blood 25(OH)D and VA concentrations with BPD, while accounting for residual confounding by strong prognostic factors—including gestational age and birth weight—a four-level multivariable binary logistic regression model was constructed, with BPD status (0 = no, 1 = yes) as the dependent variable and clinical confounders adjusted stepwise. Variable definitions and coding are provided in Table 4.

**Table 4:** Assignment of values to the variables.

Variable	Assignment
Gestational age	≥30 weeks = 0, <30 weeks = 1
body weight	≥1,500 g = 0, <1,500 g = 1
birth asphyxia	No = 0, Yes = 1
Placental abnormalities	No = 0, Yes = 1
Use of PS	No = 0, Yes = 1
Use of caffeine	No = 0, Yes = 1
Invasive mechanical ventilation	No = 0, Yes = 1
25 (OH)D	Continuous variable, unit: ng/mL
VA	Continuous variable, unit: ng/mL
Birth weight (Model 4)	Continuous variable, unit: g

The results of the multivariable binary logistic regression analysis are presented in Table 5. Model 1 revealed that gestational age <30 weeks (OR = 9.327), birth weight <1,500 g (OR = 3.394), perinatal asphyxia (OR = 2.855), and caffeine therapy (OR = 4.666) were independent risk factors for BPD (all *P* < 0.05). Model 2 showed that, without adjustment for confounders, higher umbilical cord blood 25(OH)D concentrations were associated with a reduced risk of BPD (OR = 0.868; 95% CI: 0.821–0.917; *P* < 0.001); VA concentrations were not significantly associated with BPD (*P* = 0.959). In Model 3, after adjusting for clinical confounders—including gestational age <30 weeks, birth weight <1,500 g, and caffeine therapy—umbilical cord blood 25(OH)D remained an independent protective factor against BPD (OR = 0.869; 95% CI: 0.812–0.930; *P* < 0.001);VA remained nonsignificant (*P* = 0.807). Because both gestational age and birth weight influence circulating 25(OH)D levels and are strong predictors of BPD, Model 4 additionally adjusted for birth weight as a continuous variable to further mitigate residual confounding related to prematurity. Results indicated that umbilical cord blood 25(OH)D remained independently associated with BPD (OR = 0.854; 95% CI: 0.797–0.915; *P* < 0.001).

**Table 5:** Results of MBLRM analysis.

Variable	*B*	SE	Wald	*P*	OR	95% CI
Model 1						
Gestational age <30 weeks	2.233	0.839	7.088	0.008	9.327	1.802–48.265
Birth weight <1,500 g	1.222	0.451	7.340	0.007	3.394	1.402–8.216
Birth asphyxia	1.049	0.464	5.118	0.024	2.855	1.151–7.085
Placental abnormalities	0.737	0.456	2.619	0.106	2.090	0.856–5.103
PS therapy	−0.285	0.517	0.304	0.582	0.752	0.273–2.071
Caffeine therapy	1.540	0.580	7.064	0.008	4.666	1.498–14.528
Invasive mechanical ventilation	−0.071	0.479	0.022	0.883	0.932	0.364–2.384
Model 2						
25-(OH)D(ng/mL)	−0.142	0.028	25.273	<0.001	0.868	0.821–0.917
VA (ng/mL)	0.000	0.002	0.003	0.959	1.000	0.997–1.004
Model 3						
Gestational age <30 weeks	1.844	0.907	4.131	0.042	6.319	1.068–37.388
Birth weight <1,500 g	1.785	0.524	11.619	0.001	5.960	2.135–16.633
Caffeine therapy	1.664	0.578	8.272	0.004	5.280	1.699–16.407
25-(OH)D(ng/mL)	−0.141	0.035	16.316	<0.001	0.869	0.812–0.930
VA (ng/mL)	0.000	0.002	0.060	0.807	1.000	0.997–1.004
Model 4						
Birth weight (g)	−0.005	0.001	20.715	<0.001	0.995	0.992–0.997
Caffeine therapy	1.492	0.580	6.610	<0.001	4.445	1.426–13.861
25-(OH)D(ng/mL)	−0.158	0.035	20.204	<0.001	0.854	0.797–0.915
VA (ng/mL)	0.001	0.002	0.492	0.483	1.001	0.997–1.005

Model 1 includes all prespecified clinical covariates; Model 2 estimates the crude association of umbilical cord blood biomarkers with BPD, without adjustment for confounders; Model 3 assesses the independent association of biomarkers after adjusting for clinically relevant confounders selected from Model 1—namely, gestational age <30 weeks, birth weight <1,500 g, and caffeine therapy; Model 4 further adjusts for birth weight as a continuous variable and caffeine therapy to mitigate residual confounding related to prematurity. Because gestational age and birth weight are highly collinear (variance inflation factor >10), gestational age was excluded from Model 4.

### Predictive efficacy of cord blood 25(OH)D and VA for BPD

ROC curve analysis (Table 6 and Figure 2) demonstrated that umbilical cord blood 25(OH)D concentration has strong predictive value for bronchopulmonary dysplasia (BPD) in preterm infants, with an area under the curve (AUC) of 0.824 (95% CI: 0.753–0.895). At the optimal diagnostic threshold of 22.35 ng/mL, 25(OH)D exhibited a sensitivity of 90.48%, specificity of 69.14%, negative predictive value (NPV) of 90.32%, and positive predictive value (PPV) of 69.51%. In comparison, umbilical cord blood VA concentration showed moderate predictive performance, with an AUC of 0.692 (95% CI: 0.605–0.778), sensitivity of 77.77%, specificity of 54.32%, NPV of 75.86%, and PPV of 56.98% at its optimal cut-off value of 193.50 ng/mL. Notably, the combined model incorporating both 25(OH)D and VA yielded an AUC of 0.711 (95% CI: 0.625–0.796), with corresponding diagnostic characteristics of 57.14% sensitivity, 77.77% specificity, 70.00% NPV, and 66.67% PPV.

**Table 6:** Predictive efficacy of cord blood 25(OH)D, VA on BPD.

Indicator	AUC	SE	*P*	95% CI	Sensitivity	Specificity	NPV	PPV	Cut-off value
25-(OH)D3	0.824	0.036	<0.001	0.753–0.895	90.48	69.14	90.32	69.51	22.35
VA	0.692	0.044	<0.001	0.605–0.778	77.77	54.32	75.86	56.98	193.50
Association	0.711	0.044	<0.001	0.625–0.796	57.14	77.77	70.00	66.67	–

**Figure 2:**
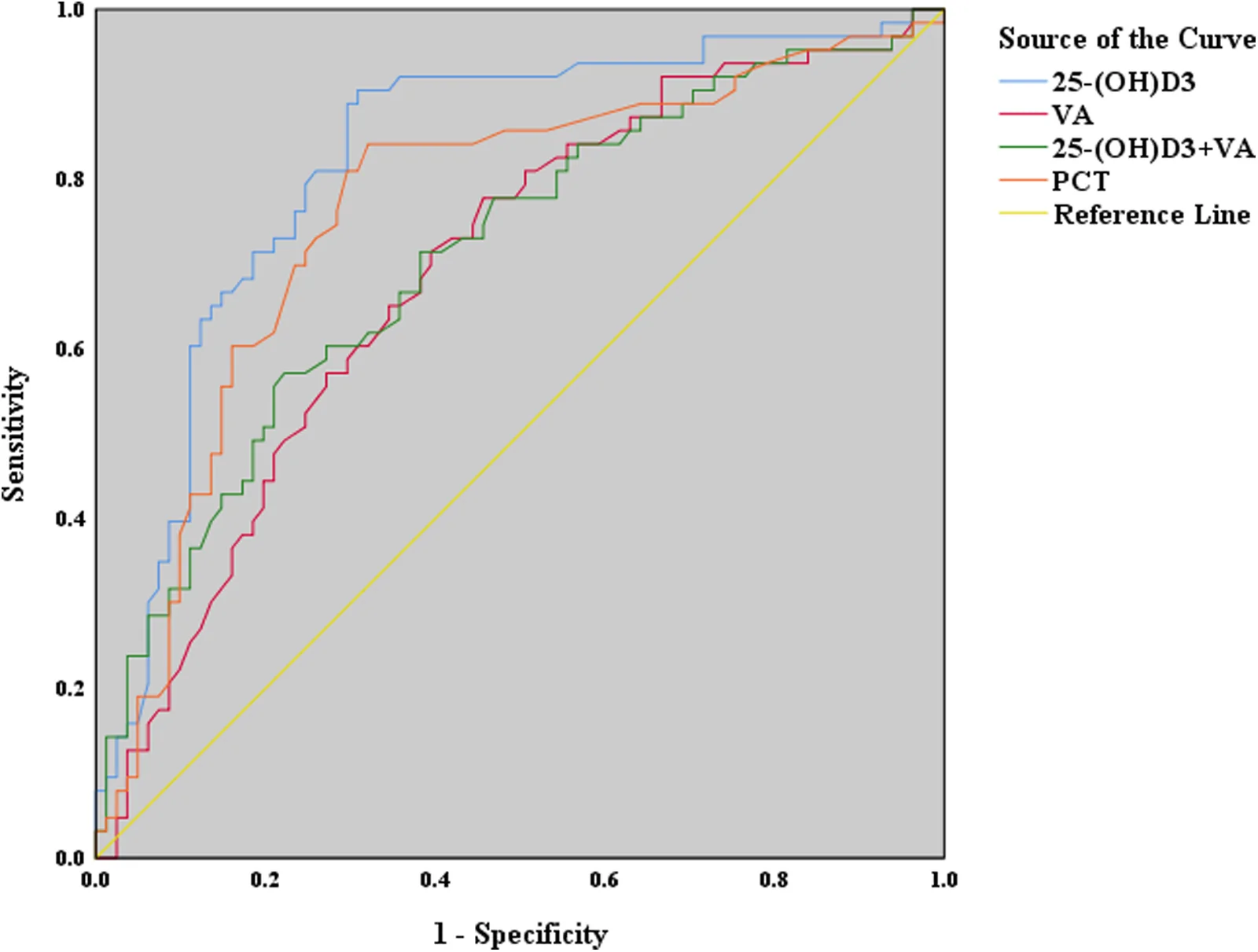
Predictive efficacy of cord blood 25(OH)D and VA for BPD.

To evaluate the incremental predictive value of umbilical cord blood 25(OH)D and VA concentrations beyond established clinical predictors—namely, gestational age and birth weight—three nested multivariable binary logistic regression models were constructed. Model 1 (the baseline clinical model) included gestational age and birth weight, yielding an area under the curve (AUC) of 0.784 (95% CI: 0.707–0.861); Model 2 added umbilical cord blood 25(OH)D concentration to the baseline model, increasing the AUC to 0.857 (95% CI: 0.792–0.922); Model 3 incorporated both umbilical cord blood 25(OH)D and VA concentrations, resulting in an AUC of 0.858 (95% CI: 0.793–0.922). The DeLong test indicated that the AUCs of Model 2 and Model 3 were each significantly greater than that of Model 1 (*P* = 0.001 and *P* = 0.002, respectively), whereas no statistically significant difference was observed between Model 2 and Model 3 (*P* = 0.772), as presented in Tables 7, 8 and Figure 3.

**Table 7:** Predictive performance of the multivariable logistic regression model.

Indicator	AUC	SE	*P*	95% CI
Model_1 (basic clinical model)	0.784	0.039	<0.001	0.707–0.861
Model_2 [basic clinical model + 25-(OH)D3]	0.857	0.033	<0.001	0.792–0.922
Model_3 [basic clinical model + 25-(OH)D3 + VA]	0.858	0.033	<0.001	0.793–0.922

**Table 8:** Pairwise comparison of ROC curves for multivariable logistic regression models (DeLong test).

First term	Second item	ΔAUC	SE	95% CI	*P* value
Model_1	Model_2	0.0712	0.0220	0.028–0.114	0.0012
Model_1	Model_3	0.0719	0.0226	0.028–0.116	0.0015
Model_2	Model_3	0.0007	0.0025	−0.004–0.006	0.7718

**Figure 3:**
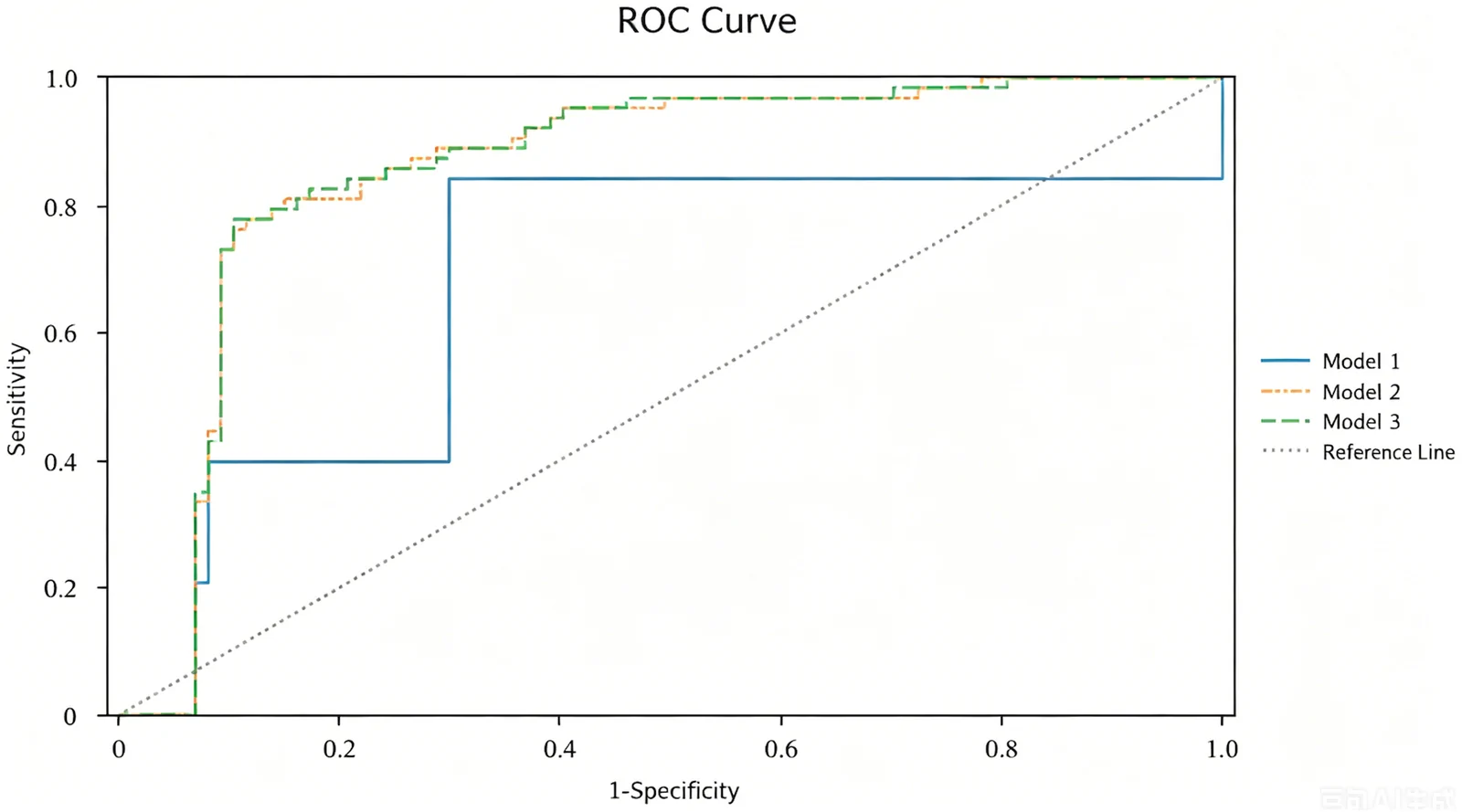
Predictive performance of the nested multivariable logistic regression model.

Further improvement in risk stratification was assessed using the net reclassification improvement (NRI) and integrated discrimination improvement (IDI). Compared with the baseline model, adding umbilical cord blood 25(OH)D concentration yielded an overall NRI of 0.875 (95% CI: 0.580–1.169; *P* < 0.001), with a positive NRI of 0.492 and a negative NRI of 0.383; the IDI was 0.158 (95% CI: 0.101–0.215; *P* < 0.001), as presented in Table 9.

**Table 9:** NRI and IDI index results.

Index	Value	SE	*z*	*P*	95% CI
NRI value	87.478%	0.150	5.823	0.000	58.035%–116.921%
NRI (gold standard = positive 1)	49.206%	0.110	4.486	0.000	27.709%–70.703%
NRI (gold standard = negative 0)	38.272%	0.103	3.728	0.000	18.152%–58.391%
IDI Value	0.158	0.029	5.413	0.000	0.101–0.215

## Discussion

Despite considerable advances in therapeutic approaches for bronchopulmonary dysplasia (BPD) (22), its incidence remains persistently high, with a troubling rise in moderate-to-severe cases (23). Clinical outcomes vary widely across the BPD severity spectrum, and higher-grade BPD is strongly associated with increased neonatal mortality and neurodevelopmental impairment (24, 25, 47). Therefore, precise stratification of disease severity is vital for early recognition of high-risk infants and for guiding targeted therapeutic interventions. However, current diagnostic criteria typically detect BPD only at later stages when irreversible pulmonary damage has already occurred, limiting the effectiveness of treatments. Consequently, identifying reliable early biomarkers for BPD prediction remains a critical research priority and clinical challenge in neonatology (25).

Vitamin D is an essential steroid hormone for humans, and vitamin D deficiency is associated with the development of multiple diseases. Cumulative evidence has confirmed that 25(OH)D plays a pivotal role in pulmonary tissue development (26, 27). Nevertheless, vitamin D deficiency among preterm infants remains a prevalent global concern (28, 29). In recent years, multiple studies have described biological links between vitamin D, lung maturation, and BPD (30–32). Preclinical investigations suggest that vitamin D may modulate lung development via regulation of alveolar and vascular morphogenesis, immune homeostasis, and tissue repair (9); however, the association between vitamin D deficiency and BPD remains controversial. In the present cohort, umbilical cord blood 25(OH)D concentrations were lower in infants with BPD relative to non-BPD controls. Within the BPD group, Grade I patients exhibited significantly higher 25-(OH)D levels than Grade III patients (*P* < 0.05), indicating an association between 25-(OH)D levels and BPD severity. These findings are broadly consistent with existing domestic and international reports. To mitigate confounding effects, hierarchical regression modelling was performed. In crude unadjusted analyses, higher cord blood 25(OH)D was associated with reduced BPD risk. After adjustment for confounders including gestational age, birth weight, and caffeine exposure, cord blood 25(OH)D remained an independent protective factor for BPD. To further account for confounding attributable to prematurity severity, birth weight was additionally entered as a continuous variable, whereas gestational age was excluded from Model 4 due to collinearity; the inverse association remained stable. As this is an observational study, only a statistical association between cord blood 25(OH)D levels and BPD can be established. No direct evidence can be inferred that 25(OH)D participates in the pathogenesis of BPD. Our findings support cord blood 25(OH)D as a promising biomarker candidate for early BPD risk assessment, yet its clinical value for intervention requires further validation in prospective interventional studies.

Vitamin A is a fat-soluble vitamin that exerts critical biological functions in fetal lung development and maturation, maintenance of respiratory epithelial integrity, and pulmonary vascular growth (33). Preterm infants are born with lower umbilical cord blood and plasma VA concentrations, as well as depleted hepatic VA stores, increasing their susceptibility to BPD and other prematurity-associated morbidities (34). VA represents one of the most extensively investigated nonenzymatic antioxidants in BPD research. Alongside the rising proportion of very low birth weight infants, its clinical use has expanded. Nevertheless, its predictive and preventive effects on BPD remain inconclusive. Although Shi et al. (35) evaluated the role of VA in children with asthma, few studies have examined its association with BPD among preterm infants. Zhang et al. (36) reported that VA deficiency may be associated with BPD under certain pathological conditions; however, the specific nature of this association requires further validation. In the present study, an inverse association was observed between umbilical cord blood VA concentration and the risk of BPD, whereas this biomarker exhibited limited utility for stratifying BPD severity. Notably, as research on VA advances, the efficacy of VA supplementation for BPD prevention has been increasingly questioned. Most existing randomized trials indicate that VA supplementation does not reduce the incidence of bronchopulmonary dysplasia (14, 37). In a 2024 publication in *The Lancet*, Sascha Meyer et al. reported findings from a multicenter, double-blind, randomized, placebo-controlled phase 3 trial enrolling 915 preterm infants with gestational age ≤32 weeks and birth weight of 400–1,000 g. Infants were randomly assigned to intervention or placebo groups. The intervention group received early high-dose enteral VA supplementation (5,000 IU/kg per day) for 28 days. This trial constitutes the largest randomized controlled trial of VA supplementation in extremely low birth weight infants to date. Contrary to findings from most prior studies, it demonstrated no benefit of high-dose VA supplementation for improving circulating VA status or reducing the incidence of moderate-to-severe BPD (13). Collectively, high-quality evidence regarding the predictive value of VA, optimal supplementation regimens and dosing, and potential synergistic applications remains insufficient. Further large-scale, prospective studies and registry-based research are warranted to address these knowledge gaps.

This study systematically assessed the predictive performance of umbilical cord blood 25(OH)D and VA concentrations for BPD in preterm infants. Univariate ROC analysis revealed that umbilical cord blood 25(OH)D concentration predicted BPD with an area under the curve (AUC) of 0.824 (95% CI: 0.753–0.895), an optimal cut-off value of 22.35 ng/mL, a sensitivity of 90.48%, and a specificity of 69.14%. These results indicate that cord blood 25(OH)D exhibits favorable predictive performance as an early screening biomarker for BPD, consistent with the findings reported by Park et al. (9). Notably, we further evaluated the incremental predictive value of 25(OH)D beyond a baseline clinical model comprising established perinatal predictors—namely, gestational age and birth weight, which achieved an AUC of 0.784. Adding umbilical cord blood 25(OH)D to this model significantly increased the AUC to 0.857 (ΔAUC = 0.073; DeLong test *P* = 0.001), while yielding a net reclassification improvement (NRI) of 0.875 (*P* < 0.001) and an integrated discrimination improvement (IDI) of 0.158 (*P* < 0.001). This finding carries important clinical implications: the predictive information captured by umbilical cord blood 25(OH)D is not merely a surrogate for gestational age and birth weight; instead, it offers additional predictive information beyond routine clinical evaluation. In clinical settings, measuring umbilical cord blood 25(OH)D may help more accurately identify preterm infants at high risk of BPD among those with comparable gestational age and birth weight, supporting decision-making for early intervention and surveillance. In contrast, the standalone AUC of umbilical cord blood vitamin A for BPD prediction was only 0.692. Incorporating vitamin A into the 25(OH)D-based clinical model did not significantly improve model discrimination (ΔAUC = 0.001, *P* = 0.772). This indicates that vitamin A has limited predictive value for BPD. Its specific role in the development of BPD, as well as the optimal timing and dosage of vitamin A supplementation, remain to be clarified in large-scale investigations. The lack of significant improvement in predictive performance after combining 25(OH)D and vitamin A also reflects that BPD pathogenesis is a complex process driven by multiple interacting factors.

Persistent inflammatory responses have been implicated in immature lung injury and aberrant pulmonary tissue and vascular development, and are considered to contribute to the development and progression of BPD (15, 25, 38, 39). IL-6, a prototypical pro-inflammatory cytokine, accelerates the progression of pulmonary inflammation (40). Cord blood IL-6 is associated with the occurrence of BPD, suggesting that it may serve as a potential biomarker for predicting BPD (41). To further verify this association, we compared cord blood IL-6 levels between infants in the BPD and control groups and found that IL-6 levels were slightly higher in the BPD group than in the control group, although the difference was not statistically significant. This finding is broadly consistent with the conclusions of a case-control study published by Wang et al. (42), which reported that IL-6 levels at birth were significantly higher in neonates with BPD than in those without BPD, although the odds ratio was very small, suggesting that elevated cord blood IL-6 levels at birth may have limited value for predicting BPD in preterm infants. However, these findings are contrary to those reported by Ambalavanan et al. (43), who measured inflammatory cytokines in peripheral blood from more than 1,000 very-low-birth-weight infants, and by Köksal et al. (44), who measured inflammatory cytokines in postnatal peripheral blood from more than 100 very-low-birth-weight infants with a gestational age <32 weeks. Possible reasons may include the relatively late onset of BPD, the considerable temporal gap between the measurement of cord blood IL-6 at birth and the diagnosis of BPD, the single-center design with a relatively small sample size, and the fact that blood was collected only at birth without serial monitoring. Future large-sample, multicenter studies are needed to further explore the association between cord blood IL-6 and BPD. In this study, we also compared IL-6 concentrations among the three BPD subgroups and found that the difference in IL-6 concentrations among the three groups of BPD neonates was statistically significant, suggesting that cord blood IL-6 levels increase with BPD severity grade and may have predictive value for BPD severity grading, consistent with current domestic and international findings (41, 42, 45).

PCT, a non-hormonally active propeptide of calcitonin, is a specific systemic inflammation biomarker. Normally, PCT exhibits minimal transplacental transfer; however, during infectious or inflammatory states, endogenous production markedly increases serum PCT levels (18). Our results showed significantly elevated PCT in BPD patients vs. controls (*P* < 0.05), with notable differences between Grade I and II cases. These findings suggest PCT may serve as an early indicator of BPD development and severity. However, the lack of significant difference between Grade I and III cases, possibly due to sample size limitations, warrants cautious interpretation.

CRP, primarily synthesized in the liver following injury or infection, serves to initiate the complement cascade, enhance macrophage phagocytosis, and modulate inflammation. CRP levels typically rise 4–6 h post-stimulus and peak around 48 h (46). In our cohort, neither IL-6 nor CRP levels differed significantly between the BPD and control groups or among severity classifications. This may reflect (1) CRP levels not elevated at birth when cord blood was collected, (2) potential false negatives in severe infections, or (3) insufficient statistical power due to limited sample size.

BPD is a multifactorial pulmonary disorder characterized by volutrauma, infection, inflammation, and impaired pulmonary repair, with a complex and incompletely elucidated pathogenesis. Elucidating prenatal, perinatal, and postnatal contributors to BPD in preterm infants is therefore essential for early prediction. Univariate analysis showed that gestational age, birth weight, birth asphyxia, placental abnormalities, PS use, caffeine therapy, and invasive mechanical ventilation were significantly associated with BPD occurrence (*P* < 0.05), consistent with previous domestic and international studies. It should be emphasized, however, that PS administration, caffeine therapy, and invasive mechanical ventilation are therapeutic and ventilatory support indicators; they represent markers of prematurity and respiratory disease severity rather than causal factors for BPD, and indication confounding may therefore be present. After multivariable adjustment, the associations of PS and invasive mechanical ventilation with BPD were no longer significant (*P* > 0.05), supporting this interpretation. Caffeine retained an independent association, although its direction and magnitude may also be influenced by treatment indications. Although this study adjusted for several confounding variables, residual confounding by disease severity cannot be fully excluded; these findings should therefore be interpreted cautiously as etiological evidence.

Umbilical cord blood (UCB) represents a non-invasive, readily obtainable biospecimen in neonates and is enriched with diverse biomarkers, making it an attractive substrate for disease prediction. In our study, umbilical artery blood was used to measure biomarker levels. From a physiological perspective, umbilical artery blood originates from the fetal systemic circulation returning to the placenta and therefore more directly reflects the fetal metabolic and inflammatory status. In contrast, umbilical vein blood is predominantly oxygenated blood returning from the placenta, and its composition is more influenced by maternal factors. Accordingly, umbilical artery blood may better represent the fetal biological state when evaluating associations between fetal lung development–related biomarkers and BPD. Regarding differences in biomarker concentrations between umbilical arterial and venous blood, existing evidence indicates that 25(OH)D is mainly supplied from the mother to the fetus, with little difference between umbilical arterial and venous levels. For certain inflammatory factors, umbilical artery blood may more directly reflect the fetal inflammatory response, whereas umbilical vein blood may also contain superimposed placental inflammatory signals. The etiology of BPD is complex, and long-term adverse outcomes are influenced by multiple factors, including genetic predisposition, infection, inflammation, and mechanical ventilation; prediction based on a single indicator is therefore insufficient. In this study, UCB was collected to measure vitamin A and vitamin D levels, in combination with serum inflammatory markers to assess inflammatory status. Compared with a single hematological parameter, this multidimensional approach may more comprehensively reflect systemic inflammatory and immune status and may offer greater value for predicting BPD and stratifying its severity.

Several methodological limitations should be acknowledged. First, as a single-center study, our findings reflect only the local situation, and their practical applicability requires validation in additional studies. Second, this study analyzed only preterm infants with a gestational age <32 weeks, and the sample size was relatively small (144 cases, of which 63 had BPD; the grade III BPD subgroup was particularly small, with only 12 cases). Consequently, the number of variables included in the multivariable model was limited, and some variables, such as birth asphyxia, may have unstable effect estimates in the fully adjusted model. Third, cord blood samples were collected only at birth, without serial monitoring of the indicators; therefore, the association between postnatal changes in these indicators and BPD occurrence could not be assessed. Fourth, data on maternal vitamin D supplementation during pregnancy and maternal blood levels are currently lacking; such data would help clarify the causes of vitamin D deficiency in preterm infants and provide a basis for developing preventive measures. Future studies should further expand the clinical sample size and conduct multicenter prospective studies for verification. Fifth, umbilical artery blood was used as the test specimen in this study, whereas most previous umbilical cord blood biomarker studies used umbilical vein blood or mixed cord blood. Although available evidence indicates that 25(OH)D concentrations differ only slightly between umbilical arterial and venous blood and that this does not affect the direction of between-group associations, the absolute concentrations of biomarkers in umbilical arterial blood may differ systematically from those in other studies. Accordingly, caution is warranted when extrapolating the present findings to umbilical vein blood or mixed cord blood samples; future studies should collect paired umbilical arterial and venous specimens to facilitate direct comparison.

## Conclusions

In conclusion, this single-center observational study identified statistical associations between cord blood 25(OH)D and VA levels, elevated IL-6 and PCT levels, and the occurrence and severity of BPD in preterm infants. Cord blood 25(OH)D < 22.35 ng/mL may serve as a valuable predictor of BPD and can be readily measured soon after birth, potentially aiding early risk identification. However, given the observational design, these findings should be interpreted as associative rather than causal. Future large-sample, multicenter prospective studies and interventional trials are warranted to confirm the predictive value and clinical utility of cord blood vitamin levels for BPD.

## Data Availability

The raw data supporting the conclusions of this article will be made available by the authors, without undue reservation.

## References

[1] Northway WH Rosan RC Porter DY. Pulmonary disease following respirator therapy of hyaline-membrane disease: bronchopulmonary dysplasia. N Engl J Med. (1967) 276:357–68. 10.1056/NEJM196702162760701 5334613

[2] Lui K Lee SK Kusuda S Adams M Vento M Reichman B. Trends in outcomes for neonates born very preterm and very low birth weight in 11 high-income countries. J Pediatr. (2019) 215:32–40.e14. 10.1016/j.jpeds.2019.08.020 31587861

[3] Durlak W Thébaud B. BPD: latest strategies of prevention and treatment. Neonatology. (2024) 121(5):596–607. 10.1159/000540002 39053447

[4] Feldman K Nitkin CR Cuna A Oschman A Truog WE Norberg M. Corticosteroid response predicts bronchopulmonary dysplasia status at 36 weeks in preterm infants treated with dexamethasone: a pilot study. Pediatr Pulmonol. (2022) 57(7):1760–9. 10.1002/ppul.25928 35434928

[5] Holzfurtner L Shahzad T Dong Y Rekers L Selting A Staude B. When inflammation meets lung development-an update on the pathogenesis of bronchopulmonary dysplasia. Mol Cell Pediatr. (2022) 9(1):7. 10.1186/s40348-022-00137-z 35445327 PMC9021337

[6] Sahni M Mowes AK. Bronchopulmonary dysplasia. In: Stern EJ, editor. StatPearls. Treasure Island (FL): StatPearls Publishing (2023). https://www.ncbi.nlm.nih.gov/books/NBK539879/. PMID: 30725945 30969701

[7] Twilhaar ES Wade RM De Kieviet JF Van Goudoever JB Van Elburg RM Oosterlaan J. Cognitive outcomes of children born extremely or very preterm since the 1990s and associated risk factors: a meta-analysis and meta-regression. Obstet Gynecol Surv. (2018) 73(10):562–3. 10.1097/01.ogx.0000547169.91377.70 PMC5875339 29459939

[8] Carbonell-Estrany X Quero J Bustos G Cotero A Doménech E Figueras-Aloy J. Rehospitalization because of respiratory syncytial virus infection in premature infants younger than 33 weeks of gestation: a prospective study. IRIS study group. Pediatr Infect Dis J. (2000) 19(7):592–7. 10.1097/00006454-200007000-00002 10917214

[9] Park HW Lim G Park Y-M Chang M Son JS Lee R. Association between vitamin D level and bronchopulmonary dysplasia: a systematic review and meta-analysis. PLoS One. (2020) 15(7):e0235332. 10.1371/journal.pone.0235332 32628705 PMC7337306

[10] Zhuxiao R Fang X Jianlan W Zhicheng Z Wei W Jiying W. Safety and feasibility of umbilical cord blood collection from preterm neonates after delayed cord clamping for the use of improving preterm complications. Am J Transl Res. (2021) 13(5):4553–60. 34150035 PMC8205698

[11] Toorell H Carlsson Y Hallberg BB O'Riordian MN Walsh BH O'Sullivan MP. Neuro-specific and immuno-inflammatory biomarkers in umbilical cord blood in neonatal hypoxic-ischemic encephalopathy. Neonatology. (2024) 121(1):25–33. 10.1159/000533473 37778335

[12] Yu H Fu J Feng Y. Utility of umbilical cord blood 25-hydroxyvitamin D levels for predicting bronchopulmonary dysplasia in preterm infants with very low and extremely low birth weight. Front Pediatr. (2022) 10:956952. 10.3389/fped.2022.956952 35989993 PMC9386287

[13] Meyer S Bay J Franz AR Ehrhardt H Klein L Petzinger J. Early postnatal high-dose fat-soluble enteral vitamin A supplementation for moderate or severe bronchopulmonary dysplasia or death in extremely low birthweight infants (NeoVitaA): a multicentre, randomised, parallel-group, double-blind, placebo-controlled, investigator-initiated phase 3 trial. Lancet Respir Med. (2024) 12(7):544–55. 10.1016/S2213-2600(24)00073-0 38643780

[14] Rakshasbhuvankar AA Pillow JJ Simmer KN Patole SK. Vitamin A supplementation in very-preterm or very-low-birth-weight infants to prevent morbidity and mortality: a systematic review and meta-analysis of randomized trials. Am J Clin Nutr. (2021) 114(6):2084–96. 10.1093/ajcn/nqab294 34582542

[15] Hsiao CC Chang JC Tsao LY Yang RC Chen HN Lee CH. Correlates of elevated interleukin-6 and 8-hydroxy-2′-deoxyguanosine levels in tracheal aspirates from very low birth weight infants who develop bronchopulmonary dysplasia. Pediatr Neonatol. (2017) 58:63–9. 10.1016/j.pedneo.2016.01.004 27321203

[16] Oor JE Daniels JMA Debets-Ossenkopp YJ De Lange-De Klerk ESM Oosterhuis JWA Dickhoff C. Bronchial colonization and complications after lung cancer surgery. Langenbecks Arch Surg. (2016) 401(6):885–92. 10.1007/s00423-016-1487-z 27485548

[17] Bose CL Dammann CE Laughon MM. Bronchopulmonary dysplasia and inflammatory biomarkers in the premature neonate. Arch Dis Child Fetal Neonatal Ed. (2008) 93(6):F455–61. 10.1136/adc.2007.121327 18676410

[18] Chauhan N Tiwari S Jain U. Potential biomarkers for effective screening of neonatal sepsis infections: an overview. Microb Pathog. (2017) 107:234–42. 10.1016/j.micpath.2017.03.042 28377234

[19] Zbyněk S Ivan B Jan Š Jan U Jaroslav F Peter K. Role of umbilical interleukin-6, procalcitonin and C-reactive protein measurement in the diagnosis of fetal inflammatory response syndrome. Ceska Gynekol. (2021) 86(2):80–5. 10.48095/cccg202180, English. 34020553

[20] Higgins RD Jobe AH Koso-Thomas M Bancalari E Viscardi RM Hartert TV. Bronchopulmonary dysplasia: executive summary of a workshop. J Pediatr. (2018) 197:300–8. 10.1016/j.jpeds.2018.01.043 29551318 PMC5970962

[21] Laboratory Medicine Branch of the Chinese Society of Geriatric Health Care, Laboratory Medicine Branch of the Chinese Society of Geriatrics. Expert consensus on standardization of 25-hydroxyvitamin D detection by liquid chromatography-tandem mass spectrometry. Chin Inspect Med J. (2021) 44(7):587595. 10.3760/cma.J.c.n114452-20201120-00846

[22] Cui X Fu J. Reinitiating lung development: a novel approach in the management of bronchopulmonary dysplasia. Respir Res. (2024) 25:384. 10.1186/s12931-024-02996-8 39449014 PMC11515458

[23] Wang J Yang G Cai Z Zhang H. Point prevalence, characteristics and treatment variations for preterm infants with bronchopulmonary dysplasia in China: a “snapshot” study. BMJ Paediatr Open. (2024) 8(1):e002878. 10.1136/bmjpo-2024-002878, Erratum in: BMJ Paediatr Open. (2024) 22;8(1):e002878corr1. doi: 10.1136/bmjpo-2024-002878corr1. 39366748 PMC11481134

[24] Li W Wang Y Song J Zhang C Xu Y Xu F. Association between bronchopulmonary dysplasia and death or neurodevelopmental impairment at 3 years in preterm infants without severe brain injury. Front Neurol. (2023) 14:1292372. 10.3389/fneur.2023.1292372 38033771 PMC10684711

[25] An N Li J Li M. Role of systemic inflammation response index and prognostic nutritional index in the prediction of moderate-to-severe bronchopulmonary dysplasia in very preterm infants. Transl Pediatr. (2025) 14(1):52–60. 10.21037/tp-24-381 39944874 PMC11811591

[26] Renqiang Y Daozhen C Qin Z Min W Yingzi M Shanyu J. Association between serum 25(OH)D levels at birth and bronchopulmonary dysplasia in preterm infants. Chin Contemp J Pediatr. (2017) 19(10):1051–5. 10.7499/j.issn.1008-8830.2017.10.004 PMC7389274 29046199

[27] Lykkedegn S Sorensen GL Beck-Nielsen SS Christesen HT. The impact of vitamin D on fetal and neonatal lung maturation. A systematic review. Am J Physiol Lung Cell Mol Physiol. (2015) 308(7):L587–602. 10.1152/ajplung.00117.2014 25595644

[28] Zhijie W Wangfang Z Fang L Peng Y Cuihua L. The vitamin D level of premature infants and correlation research on respiratory system diseases. J Mod Integ Chin West Med. (2021) 30(23):2584–7.

[29] Kim I Kim SS Song JI Yoon SH Park GY Lee YW. Association between vitamin D level at birth and respiratory morbidities in very-low-birth-weight infants. Korean J Pediatr. (2019) 62(5):166–72. 10.3345/kjp.2018.06632 30360037 PMC6528057

[30] Mandell E Seedorf GJ Ryan S Gien J Cramer SD Abman SH. Antenatal endotoxin disrupts lung vitamin D receptor and 25-hydroxyvitamin D 1alpha-hydroxylase expression in the developing rat. Am J Physiol Lung Cell Mol Physiol. (2015) 309(9):L1018–26. 10.1152/ajplung.00253.2015 26342089 PMC4628986

[31] Serce Pehlevan O Karatekin G Koksal V Benzer D Gursoy T Yavuz T. Association of vitamin D binding protein polymorphisms with bronchopulmonary dysplasia: a case-control study of gc globulin and bronchopulmonary dysplasia. J Perinatol. (2015) 35(9):763–7. 10.1038/jp.2015.58 26067474

[32] Koroglu OA Onay H Cakmak B Bilgin B Yalaz M Tunc S. Association of vitamin D receptor gene polymorphisms and bronchopulmonary dysplasia. Pediatr Res. (2014) 76(2):171–6. 10.1038/pr.2014.63 24796371

[33] Li M Cheng WX Li S Wang J Chen YR Li L. Research progress on pathophysiologic mechanisms, clinical treatment and predictive biomarkers in bronchopulmonary dysplasia: from the perspective of oxidative stress. Front Pediatr. (2025) 12:1343870. 10.3389/fped.2024.1343870 40212666 PMC11981909

[34] Shenai JP. Vitamin A supplementation in very low birth weight neonates: rationale and evidence. Pediatrics. (1999) 104(6):1369–74. 10.1542/peds.104.6.1369 10585990

[35] Meng S Jing L. Correlation analysis of vitamin A, 25-hydroxyvitamin D3 and pulmonary function levels in children with asthma of different control degrees. China Matern Child Care. (2022) 5(20):3700–3.

[36] Juan Z Libin D Yu Z. To analyze the expression characteristics of serum 25-hydroxyvitamin D, vitamin A and vitamin E levels in twin preterm infants with gestational age<32 weeks and the prognosis of the children. Chin Med Sci. (2024) 14(2):99–102.

[37] Biramalatha T Ramaswamy VV Bandyopadhyay T Somanath SH Shaik NB Pullattayil AK. Interventions to prevent bronchopulmonary dysplasia in preterm neonates: an umbrella review of systematic reviews and meta-analyses. JAMA Pediatr. (2022) 176:502–16. 10.1001/jamapediatrics.2021.6619 35226067

[38] Gilfillan M Bhandari A Bhandari V. Diagnosis and management of bronchopulmonary dysplasia. Br Med J. (2021) 375:n1974. 10.1136/bmj.n1974 34670756

[39] Schmidt AR Ramamoorthy C. Bronchopulmonary dysplasia. Paediatr Anaesth. (2022) 32:174–80. 10.1111/pan.14365 34877749

[40] Hagman C Björklund LJ Bjermer L Hansen-Pupp I Tufvesson E. Perinatal inflammation relates to early respiratory morbidity and lung function at 12 years of age in children born very preterm. Acta Paediatr. (2021) 110:2084–92. 10.1111/apa.15815 33624315

[41] Gao L Yang P Luo C Lei M Shi Z Cheng X. Machine learning predictive models for grading bronchopulmonary dysplasia: umbilical cord blood IL-6 as a biomarker. Front Pediatr. (2023) 11:1301376. 10.3389/fped.2023.1301376 38161441 PMC10757373

[42] Wang M Luo C Shi Z Cheng X Lei M Cao W. The relationship between cord blood cytokine levels and perinatal characteristics and bronchopulmonary dysplasia: a case-control study. Front Pediatr. (2022) 10:807932. 10.3389/fped.2022.807932 35463904 PMC9021742

[43] Ambalavanan N Carlo WA D'Angioct CT McDonald SA Das A Schendel D. Cytokines associated with bronchopulmonary dysplasia or death in extremely low birth weight infants. Paediatr. (2011) 123(4):1132–41. 10.1542/peds.2008-0526 PMC2903210 19336372

[44] Köksal N Kayik B Cetinkaya M Özkan H Budak F Kiliç Ş. Value of serum and bronchoalveolar fluid lavage pro- and anti-inflammatory cytokine levels for predicting bronchopulmonary dysplasia in premature infants. Eur Cytokine Netw. (2012) 23(2):29–35. 10.1684/ecn.2012.0304 22591771

[45] Yılmaz C Köksal N Özkan H Dorum BA Bağcı O. Low serum IGF-1 and increased cytokine levels in tracheal aspirate samples are associated with bronchopulmonary dysplasia. Turk J Pediatr. (2017) 59:122–9. 10.24953/turkjped.2017.02.003 29276864

[46] Xiaoli W Yingmin L Xiaoqing Y Tingting F Fei L. Correlation analysis between cord blood CRP, PCT and IL-6 and brain injury in premature infants with intrauterine infection. Chin. J. Woman Child Health Res. (2024) 35(5):71–5.

[47] Branescu I Shetty S Richards J Vladareanu S Kulkarni A. Pulmonary hypertension in preterm infants with moderate-tosevere bronchopulmonary dysplasia (BPD). Acta Paediatr. (2023) 112:1877–83. 10.1111/apa.16863 37259611

